# SARS-CoV-2 Infection and Inflammatory Response in a Twin Pregnancy

**DOI:** 10.3390/ijerph18063075

**Published:** 2021-03-17

**Authors:** Andrea Trombetta, Manola Comar, Alberto Tommasini, Melania Canton, Giuseppina Campisciano, Nunzia Zanotta, Carolina Cason, Gianpaolo Maso, Francesco Maria Risso

**Affiliations:** 1 Department of Medical, Surgical, and Health Sciences, University of Trieste, Piazzale Europa, 1, 34127 Trieste, Italy; andreamer91@live.it (A.T.); manola.comar@burlo.trieste.it (M.C.); melania.canton@libero.it (M.C.); carolina.cason@burlo.trieste.it (C.C.); 2 Institute for Maternal and Child Health “IRCCS Burlo Garofolo”, via Dell’istria 65/1, 34124 Trieste, Italy; giusi.campisciano@burlo.trieste.it (G.C.); nunzia.zanotta@burlo.trieste.it (N.Z.); gianpaolo.maso@burlo.trieste.it (G.M.); francescomaria.risso@burlo.trieste.it (F.M.R.)

**Keywords:** COVID-19, genital tract microbiome, twin pregnancy

## Abstract

There is growing literature about the SARS-CoV-2 pathogenetic effects exerted during pregnancy and whether vertical transmission or premature birth is possible. It is not well known whether changes in the immune system of pregnant women may lead to a marked susceptibility to infectious processes and the risk of adverse maternal and neonatal complications such as preterm birth, spontaneous abortion, hospitalization in an intensive care unit, transmission to the fetus or newborns, and fetal mortality are poorly understood. Along with this ongoing debate, it is not well defined whether, during pregnancy, the role of host susceptibility in producing a specific inflammatory response to SARS-CoV-2 may represent distinctive markers of risk of vertical transmission. Furthermore, SARS-CoV-2 impact on the vaginal microbiome has not yet been described, despite mounting evidence on its possible effect on the gastrointestinal microbiome and its influence on infectious diseases and preterm labor. This report describes the impact of SARS-CoV-2 on a twin pregnancy diagnosed with infection at the third trimester of gestation including tissue infections, inflammatory response, antibody production, cytokine concentration, and vaginal microbiome composition. We identified a pattern of cytokines including IL1-Ra, IL-9 G-CSF, IL-12, and IL-8 differently expressed, already associated with previously infected patients. We detected a similar concentration of almost all the cytokines tested in both twins, suggesting that the SARS-CoV-2-induced cytokine storm is not substantially impaired during the placental passage. The analysis of the vaginal microbiome did not show relevant signs of dysbiosis, similar to other healthy pregnant women and twin healthy pregnancies. The aim of this report was to analyze the immunological response against SARS-CoV-2 infection and virus tissue tropism in a twin pregnancy.

## 1. Introduction

The novel coronavirus disease 2019 (COVID-19) pandemic represents a challenge worldwide due to the significant rapidity of viral transmission and the morbidity in infected patients [1]. The public attention has recently focused on the SARS-CoV-2 pathogenetic effects exerted during pregnancy [2] and the possible fetal transmission [3,4]. Concerning the first topic, extensive population-based cohort studies have shown that seasonal influenza epidemics place pregnant women at higher risk of severe complications [5]. The existing literature reports an increased frequency of preterm births and caesarean deliveries in pregnant patients with SARS-CoV-2infection. However, generally, perinatal outcomes are characterized by mild clinical course [6]. Although several viruses are transmitted by vertical transmission, this possibility for SARS-CoV-2 has been the topic of a recent debate. Maternal COVID-19 infection in the third trimester of pregnancy is hypothesized to be associated with low transmission rates (approximately 3.2%) with no significant infant consequences [7,8]. This conclusion is consistent with recent transcriptomic data showing that the virus’s ACE-2 and TMPRSS2 proteins that enter target cells are rarely expressed on placental tissue [9].

Nevertheless, many aspects need to be carefully addressed further. During pregnancy, the role of host susceptibility in producing a specific inflammatory response to COVID-19 [10] could represent distinctive markers of risk of vertical transmission. Moreover, the role of vaginal microbiome composition in enhancing virus infection in fetal tissue has not yet been detected, although vaginal dysbiosis has been associated with increased frequency of infections and preterm labor [11]. In the current literature, there is some evidence of continuous crosstalk between the gut microbiota and the genital tract ecosystem, and specific baseline gut profiles of COVID-19 patients are associated with a more severe disease course [12].

On the other hand, despite the evidence about the host response to SARS-CoV-2 characterized by a cytokine subset including raised levels of interleukin (IL)-1β, IL-2, IL-8 IL-6, IL-10, granulocyte colony-stimulating factor, and tumor necrosis factor [13], the immune cytokines among pregnant patients with COVID-19 have recently been investigated in several singleton pregnancies [10]. In this sense, this is the first report analyzing the inflammatory response to COVID-19 in a twin pregnancy, which presents unique circulating basal plasma cytokine concentrations [14].

With this aim, this report describes the impact of COVID-19 on a twin pregnancy diagnosed with infection at the third trimester of gestation, using multiple approaches including the analysis of mother-fetal tissues for COVID-19 infection, the innate immune inflammatory response, the presence of antibodies, and the evaluation of vaginal dysbiosis by microbiome characterization. 

The patient was a 36-year-old Italian female gravida 1, para 0000 with congenital hypothyroidism and with cholestasis of pregnancy, who tested positive at the 34th gestational week and presented positive nasopharyngeal swabs, although with a very low viral load, until the day before the delivery performed at 36 + 2 with a cesarean section for transverse presentation. Both newborns did not display any perinatal complications at birth.

## 2. Material and Methods

During the last months of pregnancy, presence of SARS-CoV-2 was measured by four longitudinal nasopharyngeal swabs by real-time PCR (Neoplex™ COVID-19 Detection Kit, Genematrix, Korea). This is a qualitative test for the simultaneous detection of RdRp and N genes of SARS-CoV-2 from respiratory specimens. The test detects an internal control (IC) gene to monitor the nucleic acid isolation procedure and the possibility of PCR inhibition. The assay is performed according to the procedures suggested by the manufacturer and the guidelines of the health organization. Viral load has been measured by a home-made RT-Quantitative PCR including a standard curve targeting the N gene expressed as copy/reaction Tab 1). The vaginal microbiome composition was characterized using the Ion PGM™ System technology at SARS-CoV-2 diagnosis. The sequence data were processed by QIIME 1.8.01. as previously described [15]. After delivery, a rectal swab from the mother, nasopharyngeal swabs from the newborns, and placentas biopsies were also tested for SARS-CoV-2. In parallel, sera were obtained from the mother’s peripheral blood and the newborns’ funicular blood. Sera antibodies against SARS-CoV-2 were assessed by the Enzyme-linked immunosorbent assay (IgG seropositivity for index >1.01, Sars-Cov-2 IgG, Eurospital, Italy). In this test, specific recombinant antigens of the spike protein and of the nucleocapsid are employed. The inter and intra assay coefficient of variation was a value inferior to 10%. The inflammatory response was estimated by measuring 27 analytes including cytokines, chemokines, and growth factors (Bio-Plex Pro Human xMAP Assay, Bio-Rad, Italy) with a reported limit of detection of 1–20 pg/mL depending on the cytokine target. The total protein concentrations of samples were determined using the Bradford assay (Sigma-Aldrich, St. Louis, MO, USA) to normalize the results. All cytokine and chemokine concentrations were normalized to total protein in the sample and expressed as pg/mL. Cytokine median value from a cohort of 20 healthy pregnant women was used as reference. The signed consent to use the data for this study was obtained from the parents. The cytokine study was performed in the context of a retrospective study with the intended goal to characterize clinical manifestations, outcomes, and laboratory and imaging features of COVID-19 infection in infants, children, and pregnant women and was approved by the Institutional Review Board IRB 03/2020. A consent form was obtained directly from the patient.

## 3. Results

Table 1 describes the timing of the different sample analyses with their results. At the delivery time, the mother’s nasopharyngeal viral load dropped to a few copies, and no virus sequences were found in the placenta, rectal, and vaginal swabs. The vaginal microbiome at the time of diagnosis did not show consistent alteration of resident bacteria composition apart from the presence of a tiny percentage of *Mycoplasma, Ureaplasma,* and *Streptococcus* species. Additionally, funicular blood and nasopharyngeal samples from the newborns tested negative for COVID-19. Conversely, the inflammatory cytokine profile in sera from both the mother and funicular blood of the twins was consistent with an increased inflammatory status (Table 2). In particular, our data showed higher values of IL-1b, IL1-Ra, IL 9, IP-10, TNFa, G-CSF, and lower concentrations of IL-4, IL-8, GM-CSF, MIP1a, IL-10, IFNγ, PDGFbb, if compared with data from the same samples recovered from COVID-19 negative women at the time of delivery. However, IP-10 and IL1-Ra levels were consistently lower than severe SARS-CoV-2 infected patients [16].

IgG-specific antibodies against COVID-19 were detected in the sera and funicular blood, confirming their presence after the fifteenth day of infection, demonstrating a protective role against the virus’s vertical transmission to the twins. 

## 4. Discussion

Vertical transmission of SARS-CoV-2 occurring in utero is not yet fully defined, and fetal infection can only be definitively determined by direct demonstration of the presence of SARS-CoV-2 in fetal tissues. However, several aspects can be considered including the maternal response against viral infection. It has been postulated that SARS-CoV-2 infection can cause an imbalance in T regulatory cells [17], which are known to exert a vital role in maintaining self-tolerance, and the host microbiome [18] could influence the inhibition of the immune responses during infections [19] appropriately.

We described the impact of SARS-CoV-2 infection diagnosed at the third trimester of gestation on twin pregnancy, considering different pathogenetic features including tissue virus tropism and host immune response. The inflammatory profile and presence of antibodies have been described in sera from the mother and in funicular blood. Furthermore, the presence of dismicrobism in the vaginal microbiome composition was evaluated during the viral diagnosis.

This case report highlights the potential role of the host inflammatory response in preserving the fetus from SARS-CoV-2 vertical transmission, suggesting that pregnancy status does not influence the specific pathway of innate response against this virus.

While in this patient we did not observe vaginal dysbiosis, further work in larger cohorts are needed to delineate the interplay between SARS-CoV-2 and the vaginal microbiota in pregnancy.

This finding can be considered in light of the evidence that a status of dysbiosis in vaginal microbiome could also have a negative influence in the host response against infection during pregnancy, affecting maternal reproductive health and carrying a higher risk of adverse birth outcomes, miscarriage, and preterm birth [20].

Since no COVID-19 sequences have been detected in nasopharyngeal samples of the twins in the placenta and cord blood samples, we can argue that maternal SARS-CoV-2 IgG, together with a consistent proinflammatory response still present at the time of delivery, could have prevented the transmission of the infection to the newborn twins, as recently suggested by Flannery et al. [21].

Regarding the profiling of the mother’s immune response, we identified a pattern of cytokines including IL1-Ra, IL-9 G-CSF [22,23], IL-12, and IL-8 differently expressed, characterizing infected patients [24,25]. Moreover, the downregulated expression of GM-CSF, MIP1a, PDGFb, and IL-17 have been described in patients with severe disease [26,27]. Of note, except PDGF-bb, which was significantly lower in the second twin than in the first twin and mother, we detected a similar concentration of all the cytokines tested, suggesting that the SARS-CoV-2-induced cytokine storm is not substantially impaired during the placental passage.

Understanding the inflammatory response pattern in COVID-19 pregnancy can help to provide a new subset of the disease severity in this category of patients, especially when considering the prognostic role of immunomodulation during pregnancy. In particular, several studies have focused on the inflammatory response pattern as predictive of miscarriage [28] and the IL-10:TNF ratio has been associated with first-trimester loss [29]. As previously seen with TORCH infections [30], pregnancy complications may be, at least in part, mediated by the immune response induced by the pathogen.

Our results should be carefully considered. First, the mother was reported to have mild cholestasis of pregnancy, a condition associated with higher values of TNFa, IL1b, and Il-6 in the sera, and lower values of IL-4 and IL-10 [31,32]. Although expected, these current findings are counterbalanced by downregulation of IFNγ expression, which is reported as a hallmark of the antiviral response even in SARS-CoV-2 infection [33], being higher in pregnancy cholestasis [31] and more significant in patients infected with SARS-CoV-2 [34], as suggested in other reports [24,35]. On the other hand, IFNγ expression, along with IFN-α and IL-2 has been shown to be lower during influenza epidemic in pregnant women, when compared with nonpregnant women [36].

To our knowledge, this is the first report analyzing the immunological response against SARS-CoV-2 infection and virus tissue tropism in a twin pregnancy. Our analysis did not show any alteration in the vaginal flora, along with a strong immune response preserving the fetus from SARS-CoV-2 transmission. Further studies are needed to investigate the influence of vaginal microbiota composition on COVID-19 severity and the possible effect on the modulation of the host immune responses, as previously seen with gut microbiota [37], along with the cytokine pattern in the whole pregnancy and inflammatory markers in patients with COVID-19 and the clinical relevance on perinatal outcome in the future trend in COVID-19 pregnant women.

Elucidating the immune system’s contributions in pregnancy and fetal development can provide important insights into the pathogenesis underlying maternal and fetal diseases and suggest possible targets for therapy including modifying the composition of the microbiome of non-pregnant women infected by SARS-CoV-2.

## Figures and Tables

**Table 1:** Timing of the analysis of the different samples with results.

	Before Delivery	
Samples	Real-Time PCR SARS-CoV-2 (copies/reactions)	Microbiome analysis
* TNF swab (6/10, 34 gestational week)	POS (5.1 × 10^3^)	−
		
TNF swab (12/10, 34 + 6 gestational week)	POS (1.35 × 10^5^)	−
TNF swab (19/10, 35 + 6 gestational week)	POS (4.39 × 10^0^)	−
TNF swab (22/10, 36 + 2 gestational week)	NEG	−
Vaginal Swab	NEG	*Lactobacillus spp.* 97% *Mycoplasma hominis* 1% *Streptococcus spp.* 1% *Ureaplasma parvum serovar 6* 1%
	**After Delivery**	
Samples	Real-Time PCR SARS-CoV-2 (copies/reactions)	ELISA IgG SARS-CoV-2 (seropositivity index)
Placental biopsy I twin	NEG	−
Placental biopsy II twin	NEG	−
TNF swab I twin	NEG	−
TNF swab II twin	NEG	−
Maternal Serum	−	POS (2.032)
Umbilical blood of the first twin	−	POS (1.816)
Umbilical blood of the second twin	−	POS (1.816)

**Table 2:** Concentrations of cytokines and chemokines (pg/mL) in the maternal serum and funicular blood samples, compared to a control of 20 SARS-CoV-2 negative pregnant women.

Immune Proteins (pg/mL)	Pregnant Women SARS-CoV-2 Negative Serum (*n* = 20)	Maternal Serum	Umbilical Blood of the First Twin	Umbilical Blood of the Second Twin
**IL-1b**	n.d. (<0.47)	2.1	1.59	2.27
**IL-1ra**	152.2 (117.7 ± 172.4)	384.61	232.05	355.43
**IL-2**	13.1 (9.24 ± 16.34)	11.18	7.75	9.49
**IL-4**	7.10 (6.08 ± 8.4)	3.25	2.15	2.53
**IL-5**	n.d. (<0.58)	7.49	4.81	20.26
**IL-6**	11.42 (8.14 ± 13.61)	6.85	0.82	2.51
**IL-7**	0.6 (0.26 ± 1.06)	32.11	24.01	29.67
**IL-8**	31.91 (10.26 ± 196.6)	20.52	7.99	14.26
**IL-9**	2.01 (1.26 ± 3.34)	104.13	84.59	97.97
**IL-10**	13.7 (8.39 ± 20.81)	2.65	1.42	5.68
**IL-12(p70)**	2.37(1.08 ± 5.09)	5.09	1.68	8.28
**IL-13**	0.75 (0.49 ± 0.97)	5.12	2.79	5.12
**IL-15**	1.34 (0.42 ± 6.62)	nd	nd	nd
**IL-17**	104.18 (83.31 ± 128.3)	29.48	21.27	25.39
**Eotaxin**	3.37 (1.78 ± 8.21)	32.83	30.77	18.64
**FGF basic**	6.84 (5.76 ± 23.45)	50.68	42.59	48.1
**G-CSF**	58.36 (12.1 ± 166.7)	204.87	148.69	138.74
**GM-CSF**	44.8 (41.33 ± 80.91)	4.09	2.36	4.09
**IFN-g**	135.9 (109.6 ± 158.3)	7.15	1.4	6.65
**IP-10**	48.32 (17.98 ± 293.3)	1746.14	178.15	117.95
**MCP-1**	2.53 (2.02 ± 4.75)	30.13	13.02	16.82
**MIP-1a°**	6.80 (6.09 ± 7.51)	3.61	2.88	3.05
**PDGF-bb**	6455 (5979 ± 7582)	1752.45	1526.48	595.1
**MIP-1b**	1.47 (0.60 ± 3.24)	62.22	66.09	65.65
**RANTES**	7825 (4564 ± 5684)	8153.9	6683.47	6467.94
**TNFα**	1.84 (1.63 ± 4.69)	58.97	45.54	57.48
**VEGF**	7.41 (1.45 ± 32.68)	13.58	26.86	96.61
